# Development of a fermented quinoa‐based beverage

**DOI:** 10.1002/fsn3.436

**Published:** 2016-10-28

**Authors:** Fanny Emma Ludena Urquizo, Silvia Melissa García Torres, Tiina Tolonen, Mari Jaakkola, Maria Grazzia Pena‐Niebuhr, Atte von Wright, Ritva Repo‐Carrasco‐Valencia, Hannu Korhonen, Carme Plumed‐Ferrer

**Affiliations:** ^1^ Food BiotechnologyInstitute of Public Health and Clinical NutritionUniversity of Eastern FinlandKuopioFinland; ^2^ Department of Food TechnologyFaculty of Food EngineeringLa Molina Agrarian UniversityLimaPeru; ^3^ CEMIS‐OuluUniversity of OuluKajaaniFinland; ^4^ Green TechnologyLUKE Natural Resources Institute FinlandJokioinenFinland

**Keywords:** beverage, fermentation, quinoa, starter, varieties

## Abstract

Quinoa is a crop that originated from the Andes. It has high nutritional value, outstanding agro‐ecological adaptability, and low water requirements. Quinoa is an excellent crop alternative to help overcome food shortages, and it can also have a role in the prevention of developed world lifestyle diseases, such as type‐2 diabetes, cardiovascular diseases, osteoporosis, inflammatory and autoimmune diseases, etc. In order to expand the traditional uses of quinoa and to provide new, healthier and more nutritious food products, a fermented quinoa‐based beverage was developed. Two quinoa varieties (Rosada de Huancayo and Pasankalla) were studied. The fermentation process, viscosity, acidity, and metabolic activity during the preparation and storage of the drink were monitored, as well as the preliminary organoleptic acceptability of the product. The drink had viable and stable microbiota during the storage time and the fermentation proved to be mostly homolactic. Both quinoa varieties were suitable as base for fermented products; Pasankalla, however, has the advantage due to higher protein content, lower saponin concentration, and lower loss of viscosity during the fermentation process. These results suggest that the differences between quinoa varieties may have substantial effects on food processes and on the properties of final products. This is a factor that should be taken into account when planning novel products based on this grain.

## 1 Introduction

Quinoa (*Chenopodium quinoa* Willd.) is an ancient grain crop that originated from the Andean region of South America. Quinoa belongs to the *Chenopodiaceae* family and includes around 250 species and 3,000 varieties conserved in germplasm banks (Vega‐Galvez et al., 2010). Quinoa has an extreme agro‐ecological adaptability: It can be cultivated both in cold, highland climates, and in subtropical conditions; from sea level to above 4000 m of altitude (Miranda et al., 2012; Repo‐Carrasco, Espinoza, & Jacobsen, 2003). This property gives quinoa a good potential to be introduced around the world. Jacobsen (2003) studied the cultivation of different varieties of quinoa in North America, Africa, Asia, Australia and Europe, showing its realistic potential as a novel crop in these regions.

The extension of the global cultivation and uses of quinoa could be advisable, because the grains are highly nutritious having exceptional protein quality and a wide range of vitamins and minerals. Quinoa protein has a balanced amino acid composition being rich in essential amino acids such as lysine (5.1–6.4%) and methionine (0.4–3.1%). The total dietary fiber content of quinoa grains (average of 4.1%) compares favorably with those of wheat (2.7%) and corn (1.7%). Moreover, the amounts of calcium, magnesium, iron, and phosphorus (especially calcium and iron) are significantly higher than in most other cereals (Bhargava, Shukla, & Ohri, 2006; Repo‐Carrasco et al., 2003). Quinoa oil is rich in polyunsaturated fatty acids such as linoleic and linolenic acid, which have the potential to help in degenerative diseases such as cardiovascular diseases, cancer, inflammatory and autoimmune diseases. Quinoa grains have high concentrations of polyphenols and antioxidants such as α‐ and γ‐tocopherol—compounds suggested to have anticarcinogenic and anti‐inflammatory activities. They are also a good source of vitamin C, E, and folic acid (Bhargava et al., 2006; Jancurova, Minarovicova, & Dandar, 2009; Repo‐Carrasco et al., 2003; Schoenlechner, Wendner, Siebenhandl‐Ehn, & Berghofer, 2010). Quinoa pericarps contain up to 5% saponins, which give a bitter and astringent taste (Vega‐Galvez et al., 2010). It is necessary to wash out these undesired compounds before quinoa can be consumed.

Due to the high nutritional value, good agro‐ecological adaptability and low water requirements, quinoa has lately received a lot of attention, and several projects on a sustainable production are ongoing, to improve nutrition and to increase food security and farmer income (Giuliani, Hintermann, Rojas, & Padulosi, 2012). Quinoa could also have potential to decrease the risk of type‐2 diabetes and cardiovascular diseases, for example, hypertension (Dixit, Azar, Gardner, & Palaniappan, 2011; Ranilla, Apostolidis, Genovese, Lajolo, & Shetty, 2009). Moreover, quinoa is a suitable source of protein for vegetarians and vegans and, because it is gluten‐free, it is also an alternative cereal for people suffering from coeliac disease and gluten‐allergy problems. Thus, the use of quinoa is not only important in developing countries but also in affluent countries where there is a need to introduce new and more nutritious food products that could substitute refined carbohydrate‐rich grains such as white rice and wheat.

Quinoa has been traditionally used as cooked for salads, soups, porridges and stews, as fried patties, and drinks. Other more recent uses are as breakfast cereals, granola bars, and beer. Quinoa can also be popped and extruded, and used as “healthy” snacks. Grains can be milled into flour and used for bread‐making, pasta, biscuits, and other processed foods (Ahamed, Singhal, Kulkarni, & Pal, 1996; Bhargava et al., 2006; Diaz et al., 2013; Giuliani et al., 2012). Although there are many quinoa varieties, the variety mostly exported or cultivated in Europe nowadays is the “quinoa Real”. It is a colorless grain (creamy), with the largest grain size (2.2 mm), and preferred in the agro‐food industry around the world. However, colored quinoa (red or black) varieties are increasingly requested because of their good organoleptic potential.

In order to expand the traditional uses of quinoa and to provide new, healthier and more nutritious food products, this study aimed at developing a quinoa‐based fermented beverage. The fermentation process during the preparation of the drink was monitored as well as the metabolic activity during the fermentation and storage time. Moreover, the preliminary acceptability of the drink, by voluntary panelists, was evaluated.

## 2 Materials and Methods

### 2.1 Raw materials and nutritional composition

Two different quinoa varieties, Rosada de Huancayo (RH) and Pasankalla (PK), both cultivated in Junín, Peru, were compared during the study. PK was originally from the Andean Plateau of Puno, Peru (4,000–5,000 m of altitude), but has been adapted to lower altitude in order to improve its yield (Jancurova et al., 2009). RH has yields of 3–3.5 t/ha and a physiological maturity of 170 days. The yield for PK is a little lower, 3 t/ha, but the plant reaches its physiological maturity in only 140 days. RH is a white variety, whereas PK is a red variety.

The nutritional composition (moisture, protein, fat, crude fiber, and ash) of the two quinoa varieties was determined by standard methods (AOAC, 2005). Total carbohydrate content was calculated by subtracting the percentage sum of moisture, protein, fat, crude fiber, and ash from 100%. Saponin content was determined by the standard afrosimetric method (Koziol, 1991). Briefly, quinoa seeds (0.5 g) were mixed with 5 ml of distilled water in a capped tube and shaken vigorously for 30 s (four shakes/s, up and down). After a 30 min rest, the tube was shaken vigorously again for 30 s. This was done twice and after 5 min rest, foam height was read. The percentage of saponin was obtained from the following calculation:

$$\%\text{saponin}=0.646\left[\text{heightoffoam(cm)}\right]-0.104/{\text{sample}}^{\prime }\text{sweight(g)}*10$$

### 2.2 Bacterial strains and culture conditions

Three bacterial strains were used as starter culture for the fermentation of the quinoa‐based fermented beverage: *Lactobacillus plantarum* Q823, *Lactobacillus casei* Q11, and *Lactococcus lactis* ARH74. They were selected for their diverse technological and functional properties (Ruiz Rodríguez et al., 2016), *L. plantarum* being a potential probiotic bacterium (Vera‐Pingitore et al., 2016). *L. plantarum* Q823 and *L. casei* Q11 were isolated from quinoa grains. *L. lactis* ARH74 is a commercial strain (Valio Oy, Helsinki, Finland) well characterized as an exopolysaccharide producer (Lehto & Salminen, 1997). Overnight broth cultures of the strains were prepared the day before the fermentation and incubated at 30°C. Strains Q823 and Q11 were grown in MRS broth (Lab M, Bury, Lancashire, UK) and strain ARH74 in M17 broth (Oxoid Ltd., Hampshire, UK).

### 2.3 Samples preparation

Quinoa seeds were separated from impurities (leaves, stones, etc.) and washed thoroughly to remove saponins (foamless). The seeds were subsequently dried at 60°C for exactly 8 hr, reaching a moisture of 2.26% for PK and 3.72% for RH. After drying, quinoa seeds were milled. Quinoa flour had a final particle size of around 100 μm in both varieties.

**Figure 1:**
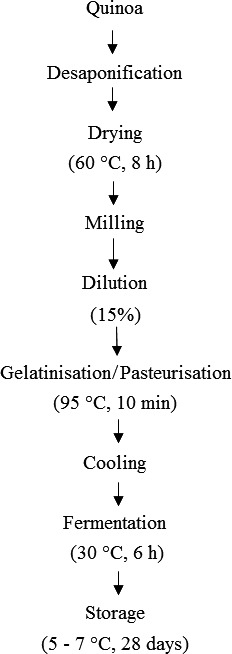
Protocol for the elaboration of the fermented quinoa‐based beverage

For the processing of the fermented quinoa‐based beverage, quinoa flours (each variety separately) were mixed with water at a concentration of 15% (w/v) (the experimentally selected minimum concentration required to prevent syneresis). The resulting quinoa slurries (250 ml) were then gelatinized/pasteurized (95°C for 10 min) and cooled down to ambient temperature before the start of fermentation. All three bacteria were inoculated at a concentration of 1% and samples were subsequently fermented for 6 hr at 30°C (Figure 1). After fermentation, 100 ml samples were stored at 5‐7°C for 28 days. Three biological replicates were done for each sample.

The pH, Total Titratable Acidity (TTA), and viscosity (Rotary Viscometer PCR‐RVI3, Model 20, UK) of the samples were monitored before and after fermentation, and at 1, 12, and 28 days of storage. TTA was titrated with 0.1 mol/L NaOH to a final pH of 8.5, detected by a pH meter. TTA was expressed as ml of 0.1 mol/L NaOH needed to achieve pH 8.5.

### 2.4 Culture viability determination

To determine the fermentation capacity of the bacterial strains and their viability during the storage time of the product, bacterial counts were measured. Samples were taken before and after fermentation, and at 1, 12 and 28 days of storage. Bacterial numbers were determined by plating 0.1 ml of three appropriate dilutions, in duplicate, on MRS agar (Lab M) plates. MRS agar plates were incubated at 30°C for 2 days and total bacterial colonies counted. Moreover, each bacterial strain was monitored separately based on their morphological colony differentiation: The colonies from *L. plantarum* Q823 are big white and shiny; the colonies from *L. casei* Q11 have irregular borders; and the colonies from *L. lactis* ARH74 are small, opaque, flat, and translucent.

### 2.5 Metabolic activity during fermentation

The metabolic activity of the fermented beverage was monitored by measuring the amounts of glucose, maltose, and sucrose, as well as the lactic, acetic, and malic acid, before and after the fermentation, and at 1, 12 and 28 days of storage. All analyses were performed with capillary zone electrophoresis (CE).

Before CE analysis of sugars, 2 g of homogenized, unfrozen (3 hr in room temperature) sample was diluted with 6–7 ml of ultrapure water and mixed thoroughly. A quantity of 100 μl of Carrez reagent I (potassium hexacyanoferrate (II) trihydrate, MERCK KGaA, Darmstadt, Germany) and Carrez reagent II (zinc sulfate heptahydrate, J.T. Baker, Deventer, Netherlands) were added to the suspension and the pH was adjusted to 7–8 with 0.1 mol/L sodium hydroxide (NaOH, J.T. Baker). The mixture was centrifuged at 3000 rpm, for 2 min at room temperature. Subsequently, the supernatant was collected and adjusted to 10 ml with ultrapure water, followed by filtration through a 0.45 μm syringe filter (VWR International, Darmstadt, Germany).

For the analysis of organic acids, the extracts were prepared in a similar fashion except that the Carrez reagents and subsequent pH adjustments were omitted.

The CE instrument used was P/ACE MDQ capillary electrophoresis system by Beckman Coulter Inc. ( Fullerton, CA, USA) with a diode array detector. The UV detection was at 270 nm and indirect detection at 232 nm for sucrose and organic acids, respectively. Sugars were measured with modified method of Rovio, Yli‐Kauhaluoma, and Siren (2007) applying buffer solution of 130 mmol/L NaOH and 36 mmol/L disodium hydrogen phosphate (Na_2_HPO_4_, MERCK) at pH 12.6. The separations were undertaken at 16°C in uncoated fused‐silica capillary with I.D. of 25 μm and total length of 40 cm (effective length of 30 cm). Separation voltage was 12 kV. Standard solutions of D(+)‐sucrose (VWR), D(+)‐maltose (Sigma‐Aldrich, Steinheim, Germany), D(+)‐glucose (VWR), and samples were introduced to capillary with pressure injection of 0.5 psi for 10 s.

Buffer system of BIS‐Tris/Pyridine dicarboxylic acid (pH 6.5) and Tris/Pyridine dicarboxylic acid (pH 8.1) by Analis along with their method (CEofix KIT, Anions 8) were used to analyze organic acids. Uncoated fused‐silica capillary with I.D. of 75 μm and total length of 60 cm (effective length of 50 cm) at a temperature of 20°C was employed. Before runs, capillary was conditioned with the buffers and flushed after with NaOH and ultrapure water. Separation was achieved with voltage of 30 kV using reversed polarity. Pressure injection of 0.5 psi for 5 s was applied for samples, and standard solutions of formic acid (ACROS Organics, Geel, Belgium), L(‐)‐malic acid (Fluka, Sigma‐Aldrich, St. Louis, USA), and acetic acid (Supelco, Bellefonte, USA) were used for quantitative analysis.

All CE analyses were performed as triplicates using L(+)‐arabinose (Fluka) as an internal standard for sugars and quinic acid (MERCK) as an internal standard for organic acids.

### 2.6 Preliminary organoleptic acceptability of the final product

The final products were evaluated by 20 volunteers. Four different products were evaluated: RH, PK, RH mixed with bilberry jam (20% bilberry and 3% of sugar), and PK mixed with dark chocolate (12%, Fazer Oy, Helsinki, Finland). The additives were chosen according to the most suitable final color. The acceptability of the appearance, color, texture, and flavor was expressed by a hedonic scale (Nicolas, Marquilly, & O'Mahony, 2010).

### 2.7 Statistical analysis

The experimental data were evaluated using analysis of variance (anova) and Tukey test, both considering a significance level of *p *<* *.05. The analyses were performed with statgraphics centurion XV software (StatPoint Technologies, Inc., Warrenton, VA). All the analyses were carried out in triplicates except for the pH and TTA (duplicates).

## 3 Results and Discussion

### 3.1 Nutritional and functional differences between quinoa varieties

The superior nutritional value of quinoa compared to many other cereals or grains is well documented (Bhargava et al., 2006; Comai et al., 2007; Vega‐Galvez et al., 2010). However, the nutritional composition of quinoa varieties may differ considerably (Repo‐Carrasco‐Valencia, Hellstrom, Pihlava, & Mattila, 2010). Although adequate comparative studies in this respect have apparently not been done yet, it is known that the nutritional composition of quinoa varieties is influenced by strong genetic variability, environmental and climatic factors (Gonzalez, Konishi, Bruno, Valoy, & Prado, 2012). In this study, the nutritional value of two quinoa varieties was studied. Quinoa PK showed significantly (*p *<* *.05) higher content of protein and fiber, and lower content of total carbohydrates and saponin than RH, whereas the fat content was similar between both the varieties (Table 1).

**Table 1:** Nutritional composition of two quinoa varieties (as g 100 g dry matter and %)

	Quinoa varieties	
	Rosada de Huancayo	Pasankalla
Moisture	10.52 ± 0.05^a^	10.61 ± 0.00^b^
Protein (*N* × 6.25)	12.75 ± 0.01^a^	14.08 ± 0.27^b^
Fat	5.18 ± 0.12^a^	5.07 ± 0.06^a^
Crude fiber	2.70 ± 0.03^a^	2.83 ± 0.07^b^
Ash	2.51 ± 0.07^a^	2.29 ± 0.05^b^
Total carbohydrates	66.35 ± 0.18^a^	65.12 ± 0.33^b^
Saponin content	0.66%^a^	0.00%^b^

The results sharing the superscript letter (within the rows) are not significantly different (*p *<* *.05).

The quinoa flours were mixed with water at a concentration of 15% (w/v). This concentration was assessed beforehand (data not shown) and it was the minimum concentration required to obtain a drink without syneresis (water phase separation) during the storage time for both varieties. However, the viscosities of the flour‐water slurries were substantially different in the beginning of the process. Quinoa's starch granules have very good pasting properties and can be used to produce high‐viscosity dough. Quinoa starch has also excellent stability under freezing and retrogradation processes (Abugoch James, 2009; Ahamed et al., 1996). However, differences between quinoa varieties are not well studied. In our study, before fermentation, the RH drink was significantly (*p *<* *.05) more viscous than the PK one, which was not expected due to the fact that higher protein in PK is known to lead to a harder and more cohesive texture (Wu, Morris, & Murphy, 2014). After fermentation, although the viscosity was reduced with both quinoa varieties, the effect was more prominent with RH. Consequently, by the end of the storage time, both quinoa varieties had the same viscosity (Table 2).

**Table 2:** Changes in the pH, total titratable acidity (TTA), and viscosity during the fermentation and storage of the quinoa‐based beverage

Days	pH				TTA^a^				Viscosity (Pas)			
	RH		PK		RH		PK		RH		PK	
	Mean	*SD*	Mean	*SD*	Mean	*SD*	Mean	*SD*	Mean	*SD*	Mean	*SD*
0^b^	6.47^a^	0.07	6.47^a^	0.07	2.20^a^	0.10	2.25^a^	0.15	53.79^a^	11.47	28.48^a^	1.89
0.25^c^	4.20^b^	0.20	4.39^b^	0.01	7.70^b^	0.20	7.30^b^	0.10	36.76^ab^	16.27	21.76^ab^	2.74
1	4.09^b^	0.11	4.28^bc^	0.08	8.25^bc^	0.25	7.90^b^	0.10	25.90^ab^	17.71	17.49^bc^	3.93
12	3.84^b^	0.16	4.14^bc^	0.04	9.25^bc^	0.25	8.35^b^	0.45	14.97^b^	10.31	12.73^c^	4.26
28	3.86^b^	0.15	3.97^c^	0.07	9.50^c^	0.50	8.60^b^	0.60	10.62^b^	5.20	10.59^c^	2.05

a TTA, ml of 0.1 mol/L NaOH per 10 g.

b Before fermentation (0 hr).

c After fermentation (6 hr).

The results sharing the superscript letter (within the columns, in lower case) are not significantly different (*p *<* *.05).

The pH decreased and TTA increased significantly in the drinks as a result of the fermentation. Fermented beverages require acid pH (4.0–4.5) in order to survive storage (Gupta, Cox, & Abu‐Ghannam, 2010). No significant change was seen during the storage time although there was a slight decrease in pH and proportional increase in TTA (Table 2). Similar behavior was observed in oat‐based fermented beverages where these factor dynamics were low (Angelov, Gotcheva, Kuncheva, & Hristozova, 2006; Gupta et al., 2010). Between quinoa varieties, there was no statistical significant difference in pH and TTA.

These results showed that not only quinoa varieties have significantly different nutritional contents but that the flours behave differently when slurried in water. These differences will be studied in more detailed in the future.

### 3.2 Bacterial strains, growth, and viability

**Figure 2:**
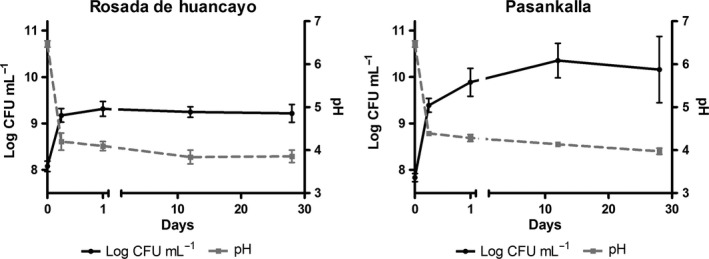
Stability and viability of the bacteria in the quinoa‐based fermented beverage

Fermentation as a food processing technique is not a novel procedure. However, fermentation with proper starter cultures can reduce the use of artificial additives such as stabilizers, thickeners, or flavors (Tiwari, Norton, & Holden, 2013). Fermentation by known lactic acid bacteria was used for the elaboration of the quinoa‐based fermented beverage reported herein. The inoculation of 1% (v/v) of each bacterial strain into the quinoa beverage resulted in initial bacterial counts of approximately log 8 CFU/ml (Figure 2). After 6 hr at 30°C, these bacteria were able to grow to a level of log 9.5 CFU/ml and decrease the pH to around 4. During the storage period, the bacteria (with the exception of ARH74) proved to be quite stable with even some increase in numbers in the PK beverage (Figure 2). After a 28‐day storage period, *L. plantarum* Q823 and *L. casei* Q11 were detected at levels more than log 9 CFU/ml. Previous studies have also reported high stability with *L. plantarum* during storage at 4°C in oat‐fermented beverages for 21 days (Angelov et al., 2006; Gupta et al., 2010). However, *L. lactis* ARH74 was lost during the storage time (data not shown). The use of PK or RH variety did not significantly affect the growth or viability of the strains.

The number of bacterial cells in food products that claim probiotic properties and the number associated with significant outcomes in clinical trials are in the range of 1–10 billion CFU per dose (Naidu, Adam, & Govender, 2002; Reid, 2005; Guarner et al. 2012). We have previously reported that *L. plantarum* Q823 can survive the passage through the human intestinal tract and thus, be a potential probiotic bacterium (Vera‐Pingitore et al., 2016). Moreover, the quinoa‐based fermented beverage developed and reported herein is able to reach a *L. plantarum* Q823 population higher than log 9 CFU/ml and thus be on the range for having probiotic activities. Consequently, the quinoa‐based fermented beverage has the potential to be used as a functional food even though the actual health benefits should be proven in a long‐term human clinical trial in order to claim that any food product has probiotic properties.

### 3.3 Metabolic activity

Fermentation is a food processing technique that can help to improve texture, structure, nutritional value, staling rate, and shelf life of food products. These qualities are associated with the production of organic acids, exopolysaccharides, aroma compounds, and antifungal compounds by lactic acid bacteria (LAB) (Wolter et al., 2014). In this study, sugars and organic acids were monitored during fermentation and storage time of the developed food product.

**Figure 3:**
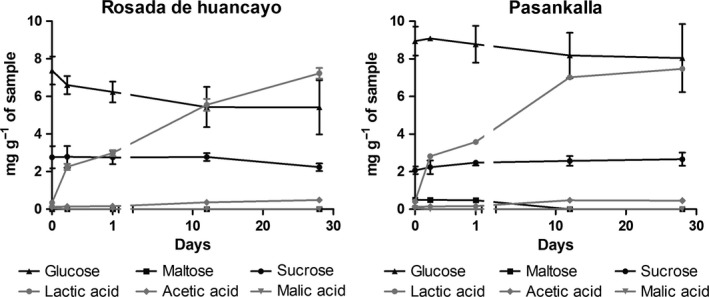
Metabolic activity in the quinoa‐based fermented beverage

The concentration of glucose, sucrose, and maltose before fermentation shows that there are significant differences between the quinoa varieties (Figure 3). Glucose concentration in RH was 7.4 mg/g, whereas in PK, it was 9.0 mg/g. Glucose concentrations decreased in both varieties during the storage time to levels of 5.2 and 8.0 mg/g in RH and PK, respectively. Sucrose and maltose were not significantly different between quinoa varieties (Figure 3).

There was a rapid increase in lactic acid concentration but not in acetic or malic acid during the fermentation (Figure 3). During the storage period, lactic acid continued to increase reaching concentrations of 7.3 and 7.5 mg/g in RH and PK, respectively.

Although endogenous enzymes from the flours can influence the metabolic activity of the drinks, the decrease in glucose and increase in lactic acid was most likely due to the activity of the lactic acid bacteria. Surprisingly, acetic acid was practically absent indicating that the fermentation of the product was mostly homolactic. It is, however, unknown the wide difference in glucose concentration by the end of the storage time in both varieties, especially because the end lactic acid concentration was almost the same. This information will be studied more deeply in the future.

### 3.4 Preliminary organoleptic acceptability of the final products

The organoleptic acceptability of four final products was evaluated (Table 3). RH and PK were not well received as such due to the characteristic sour taste. However, the acceptability of these beverages was very good when bilberries (*Vaccinium myrtillus*) and chocolate were used as flavorings. There were not significant differences in the acceptability of the product made from PK flour compared to RH although the color of the final product was dark‐brown and thus not so appealing.

**Table 3:** Preliminary organoleptic acceptability of the final product

	Rosada de Huancayo		Pasankalla	
	Natural	With bilberry	Natural	With chocolate
Overall	−2.3	1.6	−2.3	1.6
Appearance	−1.7	1.6	−0.8	1.7
Flavor	−2.5	1.3	−2.5	1.4
Odor	−0.8	1.0	−0.5	1.4
Texture	−0.7	0.3	−0.5	1.1

Nine‐point hedonic scale (−4, dislike extremely; +4, like extremely).

Values are the arithmetic average of 20 evaluators.

Although this organoleptic acceptability trial was very preliminary and it is clear that extensive research should still be done on sensory attributes, it shows that quinoa‐based drinks have the potential to be well received by consumers.

## 4 Conclusions

A fermented quinoa‐based beverage was successfully developed. Rosada de Huancayo (RH) and Pasankalla (PK) can both be considered good varieties to be used in food processing with special attention to PK due to its higher protein and lower saponin content, its lower loss of viscosity, and its higher sugar content. The development of a food product based on fermentation provided a “spoonable” beverage, without phase separation and safe low pH, and these properties were stable during the 28‐day storage period. These products could be a good source of protein, fiber, vitamins and minerals, making them not only a good snack for the coeliac and lactose‐intolerant population but also a new and exotic alternative to consumers in general. Moreover, they might support the growth and viability of probiotic bacteria, such as *L. plantarum* Q823 provided that the strain has actual long‐term health benefits.

## Conflict of Interest

The authors report no conflict of interest.

## References

[fsn3436-bib-0001] Abugoch James, L. E. (2009). Quinoa (*chenopodium quinoa* willd.): Composition, chemistry, nutritional, and functional properties. Advances in Food and Nutrition Research, 58, 1–31. 19878856 10.1016/S1043-4526(09)58001-1

[fsn3436-bib-0002] Ahamed, N. , Singhal, R. , Kulkarni, P. , & Pal, R. (1996). Physicochemical and functional properties of *Chenopodium quinoa* starch. Carbohydrate Polymers, 31, 99–103.

[fsn3436-bib-0004] Angelov, A. , Gotcheva, V. , Kuncheva, R. , & Hristozova, T. (2006). Development of a new oat‐based probiotic drink. International Journal of Food Microbiology, 112, 75–80. 16854486 10.1016/j.ijfoodmicro.2006.05.015

[fsn3436-bib-0005] AOAC . (2005). Official methods of analysis (18 edn). Gaithersburg, MD: AOAC Intl.

[fsn3436-bib-0006] Bhargava, A. , Shukla, S. , & Ohri, D. (2006). *Chenopodium quinoa* ‐ an Indian perspective. Industrial Crops and Products, 23, 73–87.

[fsn3436-bib-0007] Comai, S. , Bertazzo, A. , Bailoni, L. , Zancato, M. , Costa, C. V. L. , & Allegri, G. (2007). The content of proteic and nonproteic (free and protein‐bound) tryptophan in quinoa and cereal flours. Food Chemistry, 100, 1350–1355.

[fsn3436-bib-0008] Diaz, J. M. R. , Kirjoranta, S. , Tenitz, S. , Penttila, P. A. , Serimaa, R. , Lampi, A. , … & Jouppila, K. (2013). Use of amaranth, quinoa and kaniwa in extruded corn‐based snacks. Journal of Cereal Science, 58, 59–67.

[fsn3436-bib-0009] Dixit, A. A. , Azar, K. M. J. , Gardner, C. D. , & Palaniappan, L. P. (2011). Incorporation of whole, ancient grains into a modern Asian Indian diet to reduce the burden of chronic disease. Nutrition Reviews, 69, 479–488. 21790614 10.1111/j.1753-4887.2011.00411.x PMC3146027

[fsn3436-bib-0010] Giuliani, A. , Hintermann, F. , Rojas, W. , & Padulosi, S. (2012). Biodiversity of Andean grains: Balancing market potential and sustainable livelihoods. Rome, Italy: Biodiversity International. ISBN 13: 978‐92‐9043‐932‐5.

[fsn3436-bib-0011] Gonzalez, J. A. , Konishi, Y. , Bruno, M. , Valoy, M. , & Prado, F. E. (2012). Interrelationships among seed yield, total protein and amino acid composition of ten quinoa (*Chenopodium quinoa*) cultivars from two different agroecological regions. Journal of the Science of Food and Agriculture, 92, 1222–1229. 22002725 10.1002/jsfa.4686

[fsn3436-bib-0100] Guarner, F. , Khan, A.G. , Garisch, J. , Eliakim, R. , Gangl, A. , Thomson, A. , … & World Gastroenterology Organization (2012). “World Gastroenterology Organisation Global Guidelines: probiotics and prebiotics October 2011”, Journal of clinical gastroenterology, 46, 468–481. 22688142 10.1097/MCG.0b013e3182549092

[fsn3436-bib-0012] Gupta, S. , Cox, S. , & Abu‐Ghannam, N. (2010). Process optimization for the development of a functional beverage based on lactic acid fermentation of oats. Biochemical Engineering Journal, 52, 199–204.

[fsn3436-bib-0013] Jacobsen, S. (2003). The worldwide potential for quinoa (*Chenopodium quinoa* willd.). Food Reviews International, 19, 167–177.

[fsn3436-bib-0014] Jancurova, M. , Minarovicova, L. , & Dandar, A. (2009). Quinoa ‐ a review. Czech Journal of Food Sciences, 27, 71–79.

[fsn3436-bib-0015] Koziol, M. (1991). Afrosimetric estimation of threshold saponin concentration for bitterness in quinoa (*Chenopodium quinoa* willd). Journal of the Science of Food and Agriculture, 54, 211–219.

[fsn3436-bib-0016] Lehto, E. M. , & Salminen, S. (1997). Adhesion of two *Lactobacillus* strains, one *Lactococcus* and one *Propionibacterium* strain to cultured human intestinal caco‐2 cell line. Bioscience and Microflora, 16, 13–17.

[fsn3436-bib-0017] Miranda, M. , Vega‐Galvez, A. , Quispe‐Fuentes, I. , Jose Rodriguez, M. , Maureira, H. , & Martinez, E. A. (2012). Nutritional aspects of six quinoa (*Chenopodium quinoa* willd.) ecotypes from three geographical areas of Chile. Chilean Journal of Agricultural Research, 72, 175–181.

[fsn3436-bib-0018] Naidu, K. S. B. , Adam, J. K. , & Govender, P. (2002). The use of probiotics and safety concerns: A review. African Journal of Microbiology Research, 6, 6871–6877.

[fsn3436-bib-0019] Nicolas, L. , Marquilly, C. , & O'Mahony, M. (2010). The 9‐point hedonic scale: Are words and numbers compatible? Food Quality and Preference, 21, 1008–1015.

[fsn3436-bib-0020] Ranilla, L. G. , Apostolidis, E. , Genovese, M. I. , Lajolo, F. M. , & Shetty, K. (2009). Evaluation of indigenous grains from the Peruvian Andean region for antidiabetes and antihypertension potential using in vitro methods. Journal of Medicinal Food, 12, 704–713. 19735168 10.1089/jmf.2008.0122

[fsn3436-bib-0021] Reid, G. (2005). Food and Agricultural Organization of the United Nation and the World Health Organization. The importance of guidelines in the development and application of probiotics. Current Pharmaceutical Design, 11, 11–16. 15638748 10.2174/1381612053382395

[fsn3436-bib-0022] Repo‐Carrasco, R. , Espinoza, C. , & Jacobsen, S. (2003). Nutritional value and use of the Andean crops quinoa (*Chenopodium quinoa*) and kaniwa (*Chenopodium pallidicaule*). Food Reviews International, 19, 179–189.

[fsn3436-bib-0023] Repo‐Carrasco‐Valencia, R. , Hellstrom, J. K. , Pihlava, J. , & Mattila, P. H. (2010). Flavonoids and other phenolic compounds in Andean indigenous grains: Quinoa (*Chenopodium quinoa*), kaniwa (*Chenopodium pallidicaule*) and kiwicha (*Amaranthus caudatus*). Food Chemistry, 120, 128–133.

[fsn3436-bib-0024] Rovio, S. , Yli‐Kauhaluoma, J. , & Siren, H. (2007). Determination of neutral carbohydrates by CZE with direct UV detection. Electrophoresis, 28, 3129–3135. 17661315 10.1002/elps.200600783

[fsn3436-bib-0025] Ruiz Rodríguez, L. , Vera Pingitore, E. , Rollan, G. , Cocconcelli, P. S. , Fontana, C. , Saavedra, L. , … Hebert, E. M. (2016). Biodiversity and technological‐functional potential of lactic acid bacteria isolated from spontaneously fermented quinoa sourdoughs. Journal of Applied Microbiology., 120, 1289–1301. 26909667 10.1111/jam.13104

[fsn3436-bib-0026] Schoenlechner, R. , Wendner, M. , Siebenhandl‐Ehn, S. , & Berghofer, E. (2010). Pseudocereals as alternative sources for high folate content in staple foods. Journal of Cereal Science, 52, 475–479.

[fsn3436-bib-0027] Tiwari, B. K. , Norton, T. , & Holden, N. M. (2013). Sustainable food processing. Wiley Blackwell. ISBN: 978‐0‐470‐67223‐5.

[fsn3436-bib-0028] Vega‐Galvez, A. , Miranda, M. , Vergara, J. , Uribe, E. , Puente, L. , & Martinez, E. A. (2010). Nutrition facts and functional potential of quinoa (*Chenopodium quinoa* willd.), an ancient Andean grain: A review. Journal of the Science of Food and Agriculture, 90, 2541–2547. 20814881 10.1002/jsfa.4158

[fsn3436-bib-0029] Vera‐Pingitore, E. , Jimenez, M. E. , Dallagnol, A. , Belfiore, C. , Fontana, C. , Fontana, P. , … Plumed‐Ferrer, C. (2016). Screening and characterization of potential probiotic and starter bacteria for plant fermentations. LWT‐ Food Science and Technology, 71, 288–294.

[fsn3436-bib-0031] Wolter, A. , Hager, A. , Zannini, E. , Galle, S. , Gaenzle, M. G. , Waters, D. M. , et al. (2014). Evaluation of exopolysaccharide producing *Weissella cibaria* MG1 strain for the production of sourdough from various flours. Food Microbiology, 37, 44–50. 24230472 10.1016/j.fm.2013.06.009

[fsn3436-bib-0032] Wu, G. , Morris, C. F. , & Murphy, K. M. (2014). Evaluation of texture differences among varieties of cooked quinoa. Journal of Food Science, 79, S2337–S2345. 25308337 10.1111/1750-3841.12672

